# Basic leucine zipper ATF-like transcription factor (BATF) as a candidate marker associated with venous thromboembolism risk factors in sepsis: an integrated bioinformatics and machine learning analysis

**DOI:** 10.7717/peerj.21798

**Published:** 2026-10-20

**Authors:** Juxin Deng, Xiangyu Kuai, Zhihao Zhang, Xiuyu Ge, Zhenjie Wang, Hongchang Zhao, Lei Li

**Affiliations:** 1 Department of Emergency Surgery, The First Affiliated Hospital of Bengbu Medical University, Bengbu, China; 2 Department of Plastic Surgery and Burn, The First Affiliated Hospital of Bengbu Medical University, Bengbu, China; 3 Institute of Emergency and Critical Care Medicine, The First Affiliated Hospital of Bengbu Medical University, Bengbu, China

**Keywords:** Sepsis, Venous thromboembolism, BATF, GEO, WGCNA, Bioinformatics

## Abstract

**Background:**

Sepsis affects over 19 million people worldwide annually and is associated with an increased risk of venous thromboembolism (VTE), which significantly worsens clinical outcomes. However, current biomarkers such as D-dimer lack specificity, and the molecular signatures associated with VTE risk in sepsis remain unclear.

**Methods:**

We applied integrative transcriptomics and machine learning approaches to identify VTE-associated biomarkers in sepsis-related datasets. Weighted gene co-expression network analysis (WGCNA), differential gene expression analysis, and immune infiltration assessments were conducted using publicly available datasets. Candidate biomarkers were further evaluated using receiver operating characteristic (ROC) analysis, Cox regression, nomogram modeling, single-cell RNA sequencing, and quantitative polymerase chain reaction (qPCR) validation.

**Results:**

Basic leucine zipper ATF-like transcription factor (BATF) emerged as a top candidate biomarker, significantly associated with both sepsis and VTE-related datasets (fold change >1.5, *p* < 0.001). *BATF* expression showed consistent elevation across multiple independent cohorts and demonstrated discriminatory performance for disease- or risk-score-defined phenotypes, with area under the curve (AUC) values ranging from 0.831 to 0.953. High *BATF* expression was associated with prolonged prothrombin time and 28-day mortality in sepsis patients (*p* = 0.024). Single-cell analysis showed *BATF* enrichment in macrophages and dendritic cells.

**Conclusion:**

BATF may serve as a candidate biomarker associated with high VTE risk phenotypes and 28-day mortality in sepsis-related cohorts. However, BATF was not validated against adjudicated or imaging-confirmed VTE events in this study; therefore, it should not be interpreted as a diagnostic marker for confirmed VTE or a direct predictor of thrombosis. Prospective cohorts with adjudicated VTE outcomes are required to confirm its clinical utility.

## Introduction

Sepsis is a severe infectious disease frequently encountered in intensive care units (ICUs), representing a significant global public health challenge (Singer et al., 2016). Despite recent advancements in combating contagious agents associated with sepsis, its pathogenesis remains poorly understood. Sepsis is characterized by a complex immunological response involving a delicate balance between hyperinflammation and immunosuppression (Chalupa et al., 2011). In recent years, several biomarkers have been identified in clinical studies, such as procalcitonin (PCT), serum amyloid A (SAA) (Bengnér et al., 2021), and soluble myeloid cell triggering receptor 1 (sTREM-1) (Wang & Chen, 2011). However, due to the heterogeneity among individuals and the variations in infecting pathogens, the accurate and timely diagnosis of sepsis remains a critical challenge in clinical practice.

In the ICU setting, most patients are bedridden (Hashem, Nelliot & Needham, 2016), which significantly increases their risk of thrombus formation. Venous thromboembolism (VTE) is a serious condition characterized by coagulation disorders, leading to blood pooling in deep veins and the formation of clots (Gould et al., 2012).

Compared to the general population, sepsis patients face a markedly higher risk of developing VTE. Studies indicate that the unique inflammatory environment in sepsis contributes to this elevated risk. Sepsis-induced inflammation can result in vascular endothelium damage and coagulation disorders (Li et al., 2020). Moreover, thrombocytopenia is frequently observed in sepsis patients and has a strong association with disease progression and VTE risk (Kaplan et al., 2015; Leone, Nielsen & Russell, 2024; Peters-Sengers et al., 2022; Siniscalchi et al., 2024; Smith et al., 2014; Tabone et al., 2018). Therefore, raising awareness of the heightened risk of VTE among sepsis patients is essential for improving clinical outcomes. Implementing appropriate preventive measures is critical in reducing morbidity and mortality. Therefore, this study aimed to identify candidate biomarkers and shared transcriptomic features associated with high VTE-risk phenotypes in sepsis, to support risk stratification and future validation studies.

## Methods

### Data acquisition and processing

The datasets used in this study (GSE19151 (Lewis et al., 2011), GSE65682 (van Amstel et al., 2023), GSE48000 (Lewis et al., 2015), GSE57065 (Cazalis et al., 2014), GSE95233 (Venet et al., 2017) and GSE25504 (Dickinson et al., 2015)) were downloaded from the Gene Expression Omnibus database (https://www.ncbi.nlm.nih.gov/geo/) based on the following criteria: disease relevance, peripheral blood origin, case-control structure, and suitability for downstream analyses. Relevant platform information for each dataset was also collected. The search terms “sepsis,” “septic shock,” and “venous thromboembolism” were used to retrieve the data. All analyses were conducted using R software (version 4.4.0), which includes numerous packages suitable for bioinformatics analyses.

The dataset was processed using the following chip platforms: GSE57065, GSE25504, and GSE95233 employed the GPL570 platform (Harbig, Sprinkle & Enkemann, 2005); GSE19151 utilized GPL571 (Barrett et al., 2013); and GSE48000 employed GPL10558 (Bertone et al., 2004). These platforms correspond to the Affymetrix Human Genome U133 Plus 2.0 Array (Harbig, Sprinkle & Enkemann, 2005), Affymetrix Human Genome U133A 2.0 Array (Harbig, Sprinkle & Enkemann, 2005), Affymetrix Human Genome U133A Array (Irizarry et al., 2003), and Illumina HumanHT-12 V4.0 (Barrett et al., 2013) expression bead chip, respectively.

### Data processing and identification of differential expressed genes

The raw data for GSE57065 and GSE48000 were downloaded as Series Matrix Files from the GEO database. In R software, we annotated the normalized data by mapping probes to their corresponding gene symbols, as extracted from the platform files. A random de-duplication approach was applied to resolve probes corresponding to multiple genes.

Differentially expressed genes (DEGs) were identified using the limma R package (Ritchie et al., 2015). Statistical significance was defined by two criteria: an absolute log2 fold change (FC) greater than 1.0 and a *p*-value less than 0.05 (Ritchie et al., 2015). An absolute |log2FC| > 1, corresponding to at least a twofold expression difference, was used to prioritize biologically meaningful transcriptional changes. Overlapping DEG genes were determined based on the analytical results, and Venn diagrams were generated using the Venny online tool solely for visualization (Heberle et al., 2015). To visualize the expression patterns of DEGs, the ComplexHeatmap R package (Gu, Eils & Schlesner, 2016) was used for the GSE57065 and GSE48000 datasets.

### Construction of weighted gene co-expression network analysis about sepsis and high risk of venous thromboembolism

Weighted gene co-expression network analysis (WGCNA) is a systems biology approach used to identify gene modules that are highly correlated with diseases by constructing a gene co-expression network. We applied the WGCNA R package (Langfelder & Horvath, 2008) (version 1.72.5) to analyze peripheral blood samples from healthy individuals and patients from the GSE57065 and GSE48000 datasets. Before constructing the scale-free co-expression network, we employed the ‘goodSamplesGenes’ function (Horvath & Dong, 2008) from the WGCNA package to remove genes and samples with excessive missingness or poor quality before network construction, thereby improving the reliability of WGCNA.

After determining the optimal power, the ‘adjacency’ function was used to construct the adjacency matrix, which was subsequently transformed into a Topological Overlap Matrix (TOM) to represent network connectivity more accurately. We calculated the gene significance (GS) and module membership (MM) to assess the relationships between the identified modules and clinical traits. To visualize the overlapping genes in clinically relevant modules, we used Venn diagrams for the GSE57065 and GSE48000 datasets.

### GO and KEGG enrichment analysis

We have uploaded the filtered gene list to the GO Enrichment Tools website (https://www.bioinformatics.com.cn/), which integrates a variety R package for function analysis. For KEGG (Kyoto Encyclopedia of Genes and Genomes) (Kanehisa et al., 2025) enrichment pathway enrichment, we utilized the clusterProfiler package (Xu et al., 2024) in RStudio. To ensure accurate annotations, genes were carefully annotated using the ‘bitr’ function. Subsequently, the distribution of these enriched pathways was visualized using the ‘dotplot’ function, allowing us to effectively communicate the key findings.

### GSEA (gene set enrichment analysis)

Gene Set Enrichment Analysis (GSEA) is a powerful tool used to identify gene sets that are significantly enriched in predefined biological pathways (Subramanian et al., 2005). This method leverages differential gene expression patterns to rank genes and calculate an enrichment score for each gene set, helping to identify those consistently upregulated or downregulated across conditions.

In this study, we utilized 263 candidate genes and submitted them to an online tool (which provides integrated tools for functional enrichment analysis and graphical visualization) (Tang et al., 2023) (https://www.bioinformatics.com.cn/) to perform GSEA on both the sepsis dataset (GSE57065) and the high-risk VTE dataset (GSE48000). The aim was defined by a *p*-value of less than 0.05 and a False Discovery Rate (FDR) below 0.25, ensuring the reliability of our findings.

### Construction of PPI (protein-protein interaction networks)

To construct the protein-protein interaction (PPI) networks (Szklarczyk et al., 2023), we uploaded the list of 263 candidate genes into the STRING database (von Mering et al., 2003) (https://cn.string-db.org/). Disconnected nodes were removed, and a minimum required interaction score of 0.400 (medium confidence) was applied to ensure reliable interactions. These resulting network data were imported into Cytoscape (Shannon et al., 2003) (Version 3.10.1) for visualization and further analysis. Using the MCODE plugin (Bader & Hogue, 2003), highly interconnected gene clusters were identified to highlight potential hub modules with biological significance. For downstream hub-gene analysis, a stricter interaction confidence threshold of 0.700 was applied to focus on more robust and biologically reliable interactions, thereby reducing spurious edges and improving the interpretability of module extraction.

### Filter genes by machine learning (support vector machine and least absolute shrinkage and selection operator)

Support vector machine (SVM) (Guyon et al., 2002) and least absolute shrinkage and selection operator (LASSO) (Bodinier et al., 2023) are powerful machine-learning methods used to identify hub genes. Support vector machine recursive feature elimination (SVM-RFE) is a feature selection model designed to identify genes associated with disease diagnosis, while LASSO (Friedman, Hastie & Tibshirani, 2010) applied an adjusted parameter λ to perform variable selection. After identifying eight genes from cluster 1 using the MCODE plugin in Cytoscape (The MCODE parameters were set as follows: Degree Cutoff = 2, Node Score Cutoff = 0.2, K-Core = 2, and Max Depth = 100), we implemented the e1071 (https://cran.r-project.org/package=e1071) package (Shi et al., 2023) for SVM-RFE and used the corresponding code sourced from GitHub.

### Immune infiltration analysis

Immune infiltration analysis is essential in bioinformatics as it provides insights into the proportions of various immune cell types and the expression levels of immune cell-associated genes in specific conditions. In this study, we utilized the CIBERSORT algorithm from the IOBR package (Zeng et al., 2021) to perform immune infiltration analysis. The input data for this analysis were the pre-processed matrix files from GSE57065 and GSE48000. For data visualization, we used the linkET (Huang, 2021) package, which integrates functions from both the purrr (https://CRAN.R-project.org/package=purrr, A complete and consistent functional programming toolkit for R.) and ComplexHeatmap package (Gu, 2022), allowing us to effectively present and interpret the results.

### Clinical correlation analysis

To assess the clinical relevance of the hub gene, sepsis patients were stratified based on clinical metadata available in the GSE65682 dataset. We performed both univariate and multivariate Cox regression analyses using the survival and survminer package (Ananth & Kleinbaum, 1997; Kassambara & Biecek, 2024), enabling us to predict 28-day survival for ICU patients. Furthermore, we categorized the hub gene into high and low-expression groups and conducted survival analysis to evaluate the prognostic significance of the genes in sepsis at high risk for VTE.

### scRNA-seq analysis

To provide cellular context for the bulk transcriptomic findings, single-cell RNA sequencing (scRNA-seq) analysis was performed to identify the immune cell populations associated with *BATF* expression. The peripheral blood sepsis dataset (GSE167363) was obtained from the GEO database and analyzed using the ‘Seurat’ package (Hao et al., 2024). During quality control, cells with fewer than 200 detected genes or mitochondrial transcript content greater than 10% were excluded to remove low-quality, dying, or apoptotic cells. After normalization and dimensionality reduction, two-dimensional cell embedding was generated using the ‘RunUMAP’ function. Cell clustering was performed using ‘FindClusters’ function based on the same principal components used for ‘RunUMAP’ visualization.

### Cell culture and sepsis model construction

Cell culture and inflammatory stimulation were performed with reference to our previous protocol (Cheng et al., 2025), with modifications for the present study. THP-1 cells and human umbilical vein endothelial cells (HUVEC) were obtained from the American Type Culture Collection (ATCC). THP-1 cells were maintained in RPMI-1640 medium containing 10% fetal bovine serum (FBS; HyClone, Logan, UT, USA) and 100 U/mL penicillin–streptomycin at 37 °C in a humidified incubator with 5% CO_2_. To generate a macrophage-like inflammatory model, THP-1 monocytes were exposed to 100 nM phorbol 12-myristate 13-acetate (PMA) for 48 h and subsequently challenged with 1 µg/mL lipopolysaccharide (LPS) for 24 h. In parallel, HUVECs were treated with LPS for 24 h to establish an endothelial inflammatory model relevant to thromboinflammatory responses.

### Clinical sample collection

Peripheral blood samples were collected from sepsis patients diagnosed according to the Sepsis-3 criteria at The First Affiliated Hospital of Bengbu Medical University and from healthy volunteers as controls (Table S1). For the high-risk VTE group, peripheral blood samples were obtained from sepsis patients classified as having a high risk of VTE based on clinical risk stratification (*e.g*., Caprini score ≥5), without clinically adjudicated or imaging-confirmed thrombotic events at the time of sampling. Healthy volunteers served as controls (Table S2). Total RNA was extracted from whole blood using the TRIzol reagent (R0016; Beyotime, Shanghai, China) following the manufacturer’s protocol (Chomczynski & Sacchi, 1987). The study was approved by the Ethics Committee of Clinical Medical Research of the First Affiliated Hospital of Bengbu Medical University (Ethical approval number: [2024] KY018), and written Informed consent was obtained from all participants.

### RNA extraction and quantitative PCR analysis

The study included three groups: sepsis patients, sepsis patients at high risk of venous thromboembolism (VTE), and healthy controls. Quantitative polymerase chain reaction (qPCR) validation was performed to compare *BATF* expression levels among the groups. Peripheral blood samples from sepsis and high-risk VTE patients (~200 μL per extraction) and LPS-stimulated THP-1-derived macrophages (~1 × 10^6^ cells) were used. All samples were processed within 2 h after collection, immediately lysed in TRIzol reagent, and stored at –80 °C. No tissue fixation was performed. Total RNA was subsequently extracted using TRIzol reagent (Invitrogen, Carlsbad, CA, USA) according to the manufacturer’s protocol (Chomczynski & Sacchi, 1987) (Invitrogen, Carlsbad, CA, USA). RNA quantity was determined using a NanoDrop 2000 spectrophotometer (Thermo Fisher Scientific, Waltham, MA, USA), and samples with A260/A280 ratios between 1.9 and 2.1 were used for downstream analysis. Reverse transcription was performed using a cDNA synthesis kit (AT-341; TransGen Biotech, Beijing, China), RNA samples were treated with DNase during the reverse transcription process, as the TransScript^®^ All-in-One First-Strand cDNA Synthesis Kit includes a genomic DNA removal step to eliminate residual DNA contamination, Reverse transcription was performed using the TransScript^®^ All-in-One First-Strand cDNA Synthesis Kit (AT-341; TransGen Biotech, Beijing, China) according to the manufacturer’s protocol (Krug & Berger, 1987). A total of 200 ng of RNA was used in a 20 μL reaction volume, containing TransScript^®^ RT/RI Enzyme Mix, buffer, and oligo(dT)18. The reaction was incubated at 42 °C for 30 min, followed by 85 °C for 5 s to terminate the reaction. qPCR analysis was conducted with SYBR Green-based detection (AQ-601; TransGen Biotech, Beijing, China) on a Real-Time PCR system (LightCycler 480 Real-Time PCR System (Roche, Basel, Switzerland)). Gene-specific primers of *BATF* (110 bp) amplicon were F: TATTGCCGCCCAGAAGAGC; R: GCTTGATCTCCTTGCGTAGAG. RPLP0 were F: AGCCCAGAACACTGGTCTC; R: ACTCAGGATTTCAATGGTGCC. GAPDH (F: GTCTCCTCTGACTTCAACAGCG; R: ACCACCCTGTTGCTGTAGCCAA), results were consistent when normalized to both RPLP0 and GAPDH; GAPDH was used for final normalization. Each reaction was performed in triplicate in a 20 μL total volume containing 10 μL SYBR mix, 0.4 μL of each primer (10 μM), and 2 μL of cDNA template. The thermal cycling conditions were 95 °C for 30 s, followed by 40 cycles of 95 °C for 5 s and 60 °C for 30 s. Melting curve analysis confirmed the specificity of amplification. The 2^−ΔΔCt^ method was employed to calculate the relative expression level of BATF, with GAPDH as the final internal control. All experiments were repeated at least three times. qPCR data were expressed as mean ± standard deviation (SD) from at least three independent experiments. Comparisons between two groups were analyzed using the unpaired Student’s t-test, whereas comparisons among three groups were analyzed using one-way analysis of variance (ANOVA) followed by Tukey’s *post hoc* test.

Statistical significance was defined as *p* < 0.05. All graphs were generated using GraphPad Prism 8 unless otherwise stated.

### BATF knockdown and Western blot analysis

To evaluate the effect of BATF knockdown on inflammatory signaling at the protein level, small interfering RNA (siRNA) targeting *BATF*, and a negative control siRNA were transfected into THP-1 cells and HUVECs using Lipofectamine 3000 (Catalog No. L3000015; Thermo fisher Scientific, Waltham, MA, USA) according to the manufacturer’s instructions. After transfection for 24 h, cells were stimulated with lipopolysaccharide (LPS, 100 ng/mL) for the indicated time. These *in vitro* models represent inflammatory and thromboinflammatory activation rather than direct thrombosis models.

Total cellular proteins were extracted using RIPA lysis buffer supplemented with protease inhibitor cocktail. Protein concentrations were determined using a BCA protein assay kit. Equal amounts of protein (20–30 μg) were separated by SDS–PAGE and transferred onto PVDF membranes. The membranes were blocked with 5% non-fat milk in TBST for 1 h at room temperature and incubated overnight at 4 °C with primary antibodies against BATF (Catalog No. 13507-1-AP; Proteintech, Wuhan, China) and IL6 (Catalog No. A27935; ABclonal, Wuhan, China). After washing, membranes were incubated with the corresponding HRP-conjugated secondary antibodies for 1 h at room temperature.

Protein bands were visualized using an enhanced chemiluminescence (ECL) detection system, and images were captured using a chemiluminescence imaging system. β-actin was used as the loading control to normalize protein expression levels.

### Overall analytical workflow and rationale

This study employed a stepwise integrative analytical strategy to systematically identify and validate candidate biomarkers associated with VTE risk-related transcriptomic patterns in sepsis.

First, differential expression analysis was performed to identify genes significantly altered in sepsis and VTE-risk cohorts. Second, WGCNA was used to identify gene modules associated with disease-related phenotypes, enabling the identification of co-regulated gene clusters rather than individual genes alone. Third, overlapping genes identified from differential expression and WGCNA analyses were subjected to functional enrichment analysis (GO, KEGG, and GSEA) to characterize their biological relevance and pathway associations. Fourth, PPI (protein–protein interaction) network analysis and module detection were conducted to identify highly interconnected gene clusters, reflecting potential biological hubs. Fifth, machine learning algorithms LASSO and SVM-RFE were applied to prioritize the most informative candidate biomarkers with diagnostic relevance. Sixth, immune infiltration analysis and single-cell RNA sequencing (scRNA-seq) were used to provide cellular-level context and identify immune cell populations associated with candidate gene expression. Finally, clinical relevance was validated *via* clinical correlation and qPCR of *BATF*. To provide mechanistic support, Western blot and siRNA-mediated knockdown were performed to evaluate the functional impact of BATF on inflammatory signaling (*e.g*., IL6 expression).

This stepwise approach allowed progressive refinement from global transcriptomic alterations to biologically and clinically relevant candidate biomarkers.

## Result

### Shared gene signatures link sepsis and VTE in transcriptomic profiles

In the GSE57065 dataset for sepsis, we analyzed samples from 28 sepsis patients and 25 healthy controls. A total of 1,174 differentially expressed genes were identified, comprising 583 upregulated and 591 downregulated genes (Figs. 1A, 1B). Similarly, in the GSE48000 dataset, which includes 25 control samples and 41 patients at high risk for venous thromboembolism (VTE), we identified 3,404 DEGs. Among these, 2,504 genes were upregulated and 900 were downregulated (Figs. 1C, 1D). A Venn diagram was used to highlight the overlap between the two datasets, revealing 166 overlapping genes (Fig. 1E). These shared DEGs may represent critical molecular signatures associated with both sepsis and a high risk of VTE.

**Figure 1:**
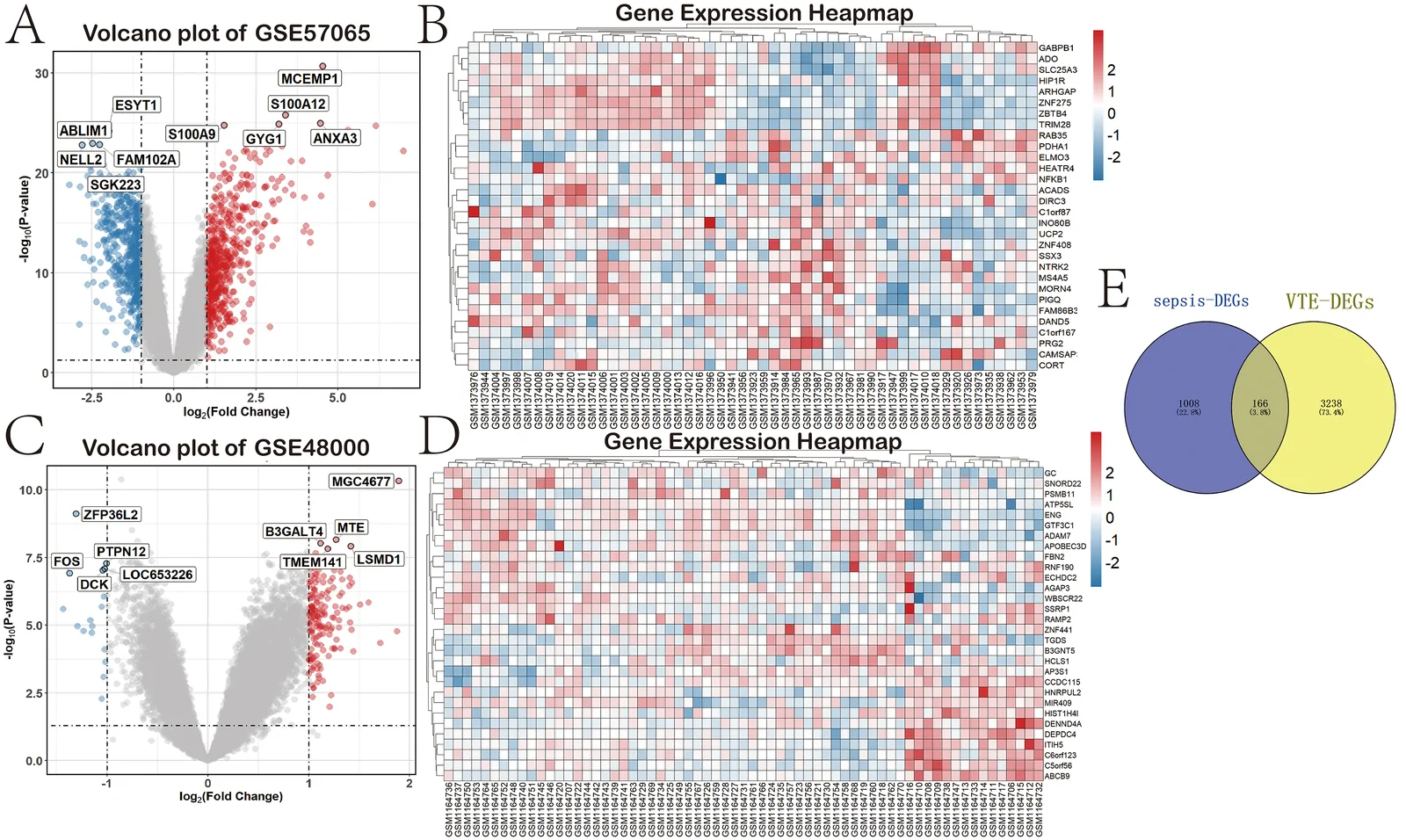
Identify the DEGs in GSE48000 and GSE57065. (A, B) The volcano plot and the heatmap of GSE57065. (C, D) The volcano plot and the heatmap of GSE48000. (E) The Venn plot of overlapping genes.

### Co-expressed genes enriched in immune activation and coagulation cascades

We employed WGCNA to identify genes that correlate with clinical traits in the sepsis dataset (GSE57065) and the high-risk VTE dataset GSE48000. No significant outlier samples were detected in either dataset. Following the WGCNA methodology’s recommendation, the soft threshold power was selected based on the scale-free topology criterion. The optimal β value was selected when the scale-free topology fit index reached approximately 0.8 while maintaining sufficient mean connectivity. Accordingly, the soft threshold power was set to four for the GSE57065 dataset and nine for the GSE48000 dataset (Figs. 2A, 2B). Taking advantage of the modules’ interrelated similarities, we identified 16 modules in the GSE57065 dataset and 21 modules in the GSE48000 dataset (Figs. 2C, 2D). Module-trait correlations were then analyzed, revealing that the brown module in the GSE57065 dataset showed the strongest association with clinical traits r = 0.84; Fig. 2E. Similarly, the dark slate blue module in the GSE48000 dataset, displayed the highest correlation with clinical traits r = 0.56; Fig. 2F. Further analysis demonstrated a strong relationship between gene significance (GS) and module membership (MM) within these key modules, with correlation coefficients of 0.93 for the sepsis and 0.84 for the high-risk VTE dataset. These findings highlight the significant role of these genes in disease progression.

**Figure 2:**
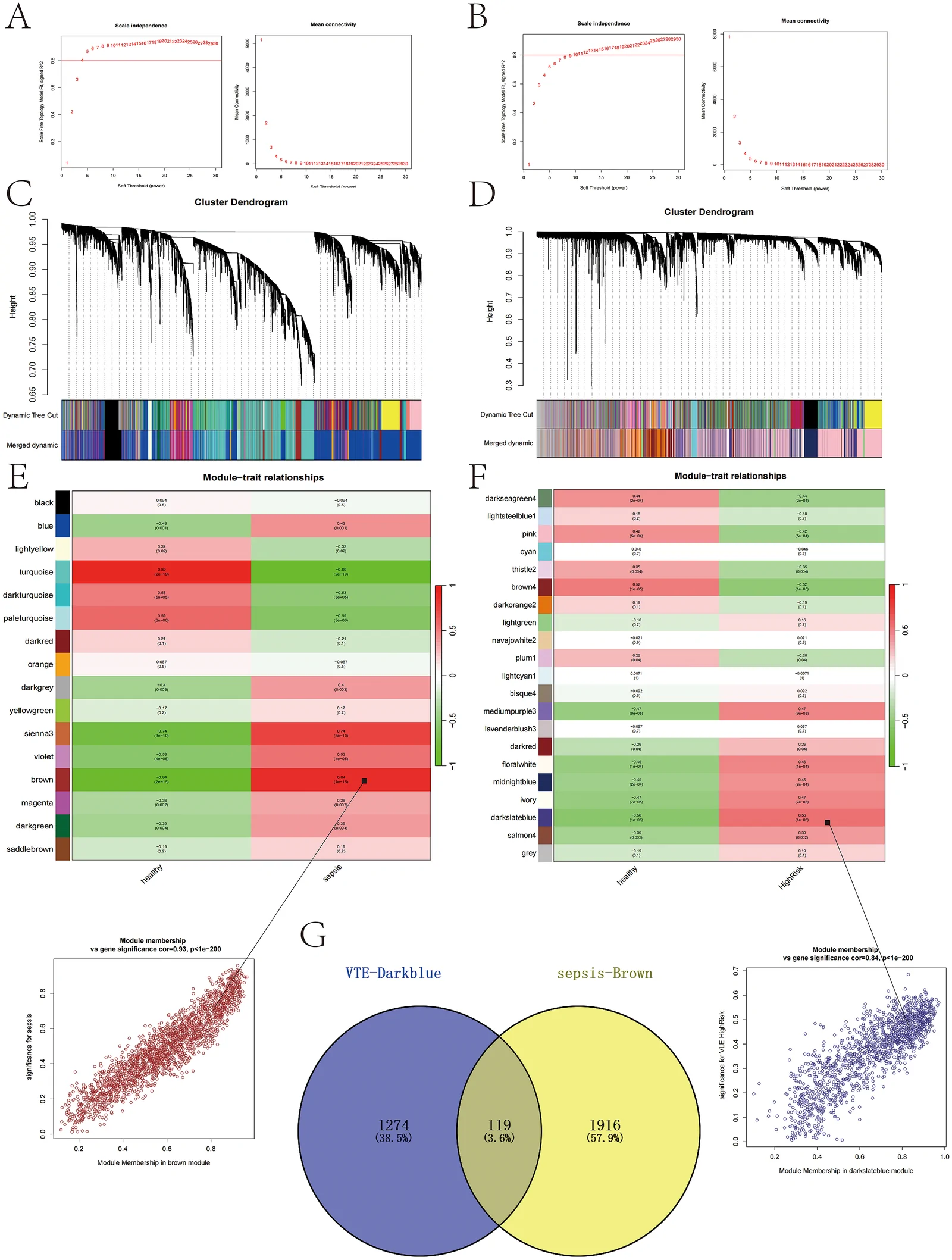
The result of the WGCNA. (A, B) The mean connectivity for scale independence and soft threshold in sepsis dataset and VTE dataset respectively. (C, D) Clustering plot of sepsis and VTE. (E, F) The diagram of the correlation analysis that between gene modules with clinical phenotypes in VTE and sepsis datasets. The brown module and Darkblue module were the best positive correlation in twice diseases respectively. (G) The Venn diagram of the overlapping genes which between dark blue module and brown module.

Finally, we identified 119 overlapping genes between the clinically significant modules in the sepsis and high-risk VTE datasets. These shared genes may reflect common transcriptomic programs associated with sepsis and high VTE-risk phenotypes (Fig. 2G).

### Identification and functional analysis of hub genes linking sepsis and VTE

We identified 166 overlapping genes from the differentially expressed genes (DEGs) and 119 co-occurring genes within the two disease-associated modules described earlier. To ensure comprehensiveness, we combined the genes through differential analysis and WGCNA results, removing duplicates. This process yielded a total of 263 co-driver genes, which may play pivotal roles in the key molecular pathways underlying high-risk venous thromboembolism (VTE) in sepsis datasets.

To explore the biological significance of these genes, we conducted Gene Ontology (GO) and Kyoto Encyclopedia of Genes and Genomes (KEGG) pathway enrichment analyses. GO analysis revealed that these genes are involved in critical biological processes, including immune cell differentiation and activation, specific granules production, cytokine activity, protein serine/threonine kinase activity, and organelles’ function (Fig. 3A). KEGG pathway analysis highlighted their involvement in infectious, immune, and inflammatory pathways, including Kaposi sarcoma-associated herpesvirus infection, PD-L1 expression and the PD-1 checkpoint pathway in cancer, and Th17, Th1, and Th2 cell differentiation pathways (Fig. 3B).

**Figure 3:**
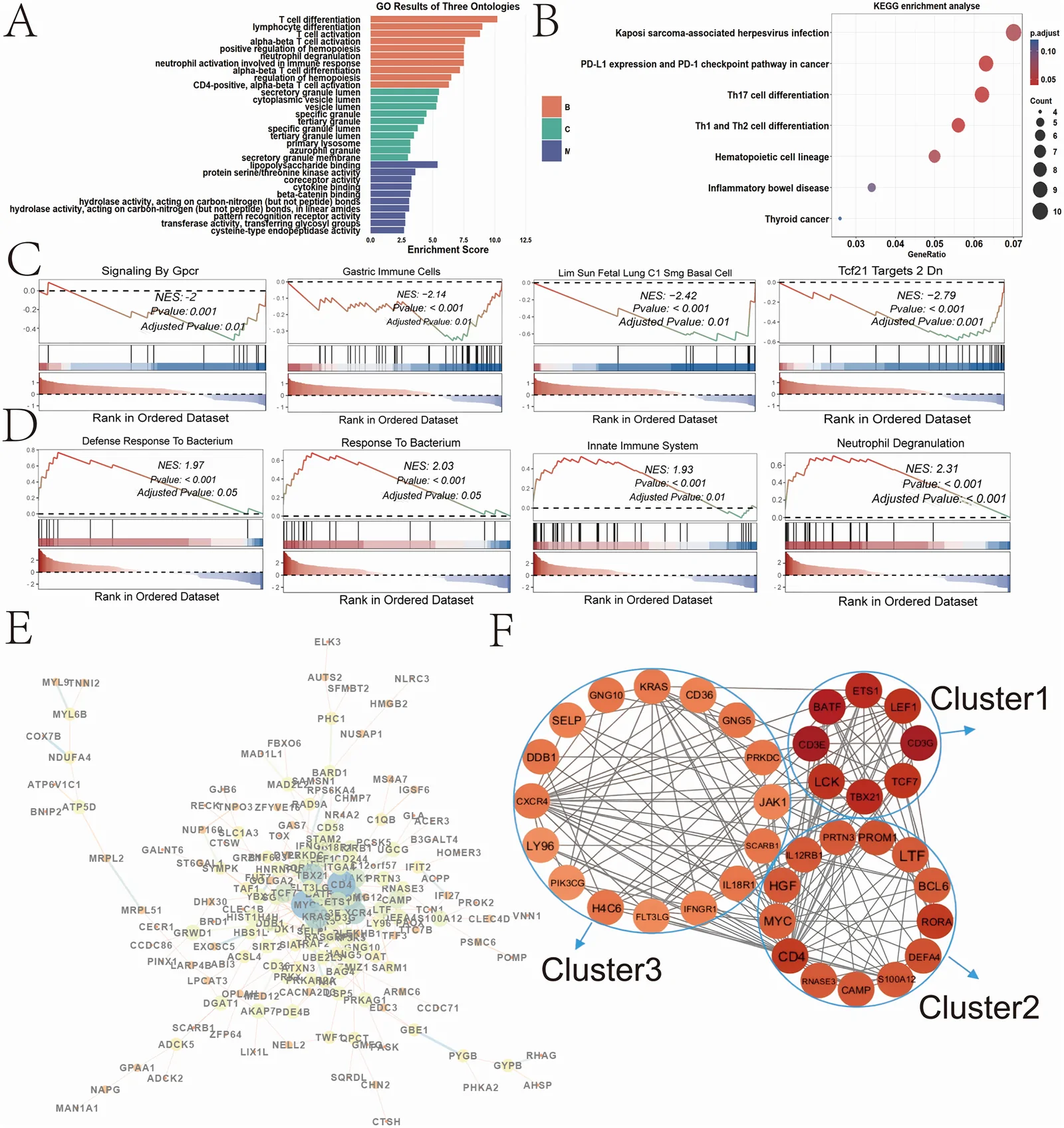
Function and pathway enrichment of co-driver genes. (A, B) Go and KEGG enrichment analysis. (C) GSEA result of GSE48000 (D) GSEA result of GSE57065. (E) PPI (protein and protein network) form STRING database. (F) Three clusters of HUB genes.

To mitigate potential redundancy in enrichment results, we performed Gene Set Enrichment Analysis (GSEA) on the co-driver genes from both the VTE and sepsis datasets. The results revealed that the co-driver genes were negatively associated with immune cells and pathways in the VTE dataset, such as signaling by GPCRs, gastric immune cells, lung basal cells, Tcf Target 2 Dn, and Kaposi sarcoma-associated herpesvirus infection (Fig. 3C). Conversely, in the sepsis dataset, the co-driver genes demonstrated a positive association with pathways involved in infectious and immune processes, including response to bacteria, defense response to bacteria, the innate immune system, and neutrophil degranulation (Fig. 3D). These findings suggest that co-driver genes are associated with distinct immune and inflammatory programs in VTE-related and sepsis-related contexts. To further explore the function of the co-driver genes and identify the potential hub genes, we input the 263 co-driver genes into the STRING database with a high confidence threshold of 0.700 (Fig. 3E). Following this, we excluded independent gene clusters using Cytoscape and organized the remaining genes into three modules (clusters 1–3) with the MCODE plugin, resulting in 35 hub genes (Fig. 3F). Among these, we identified *CD3E, CD3G, LEF1, BATF, TBX21, TCF7, ETS1*, and *LCK* as candidate hub genes, as they scored highly in cluster 1, suggesting their pivotal roles in transcriptomic programs associated with high-risk VTE phenotypes in sepsis. These genes are mainly involved in lymphocyte activation, T-cell differentiation, and immune signaling, suggesting that this module reflects immune-regulatory processes relevant to sepsis and high VTE-risk phenotypes. Among them, *BATF* was selected for downstream analysis because it was the only gene consistently prioritized across machine-learning methods and multiple validation datasets.

### *BATF* validated as a candidate marker associated with sepsis and VTE risk

To refine the identification of hub genes in cluster 1 relevant to diagnosis, we applied machine learning algorithms to analyze the VTE and sepsis datasets. Among the eight candidate genes filtered by MCODE in Cytoscape, we employed LASSO regression and SVM-RFE for feature selection.

In the GSE48000 dataset, LASSO identified six genes as significant candidates (Figs. 4A and 4B), while the SVM-RFE method narrowed the selection down to one gene (Fig. 4C). Similarly, in the GSE57065 dataset, the LASSO approach screened four genes (Figs. 4D and 4E), whereas SVM-RFE highlighted nine genes (Fig. 4F).

**Figure 4:**
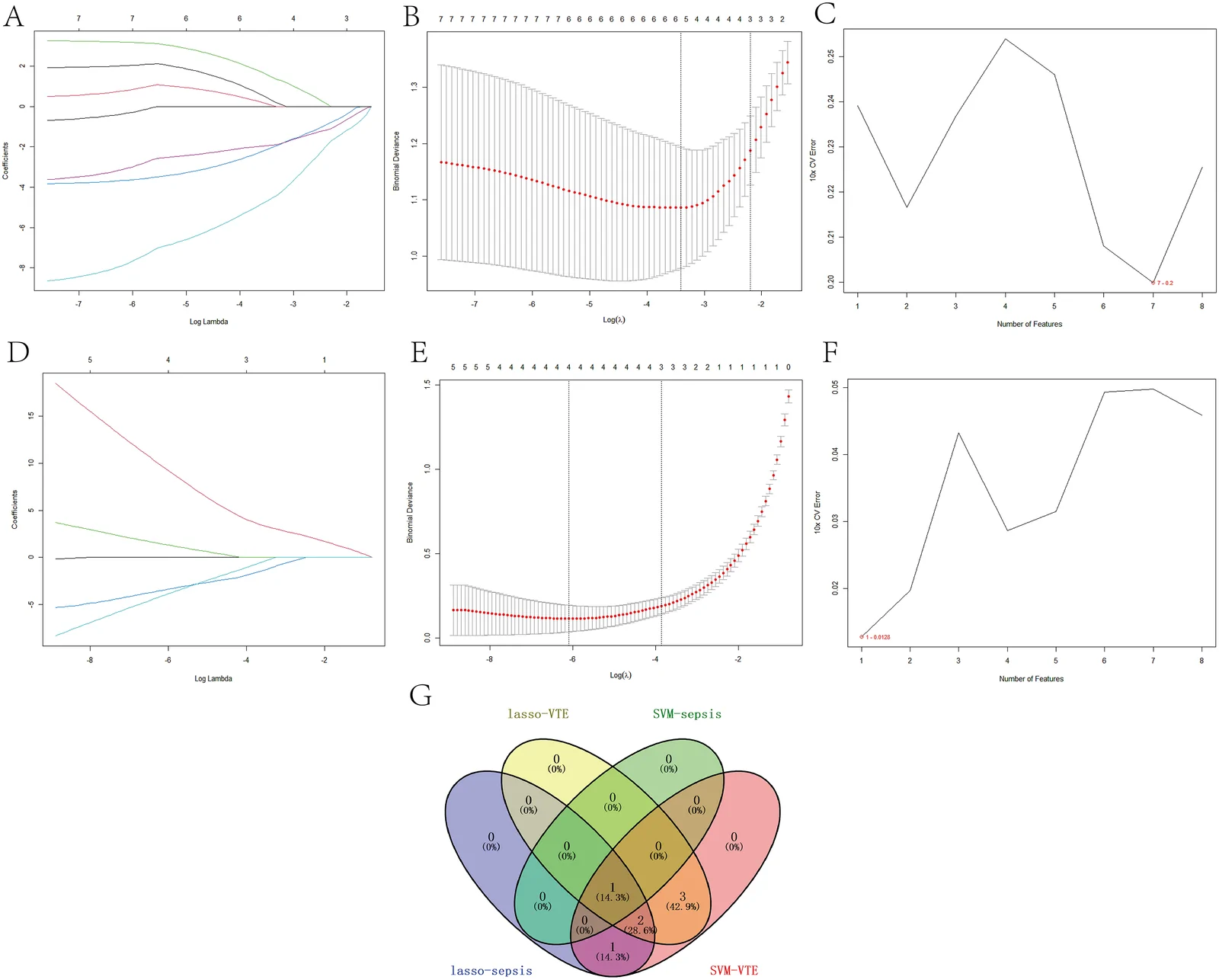
Identify the hub genes by LASSO and SVM. (A, B, D, E) GSE57065 and GSE48000 by LASSO regression analysis. (C, F) SVM-RFE analysis of sepsis and VTE dataset. (G) Venn diagram of overlapping genes filtering by two kinds of machine learning method.

By intersecting the genes identified across the various methods and datasets, *BATF* emerged as the only overlapping candidate (Fig. 4G). This finding supports *BATF* as a candidate biomarker associated with sepsis and high VTE-risk phenotypes.

### *BATF* shows discriminatory performance across independent sepsis- and VTE-related cohorts

To evaluate the discriminatory performance of BATF for disease- or risk-score defined phenotypes across different datasets, we also employed ROC curves (Figs. 5A–5D). We assessed *BATF*'s performance in high VTE-risk related datasets (GSE48000 and GSE19151) and sepsis datasets (GSE57065 and GSE25504). To further validate its classification performance in sepsis datasets with a high risk of VTE, we analyzed the GSE65682 dataset (Fig. 5E), which included sepsis samples with a stringent diagnosis and a Caprini score greater than 5, indicating an elevated VTE risk.

**Figure 5:**
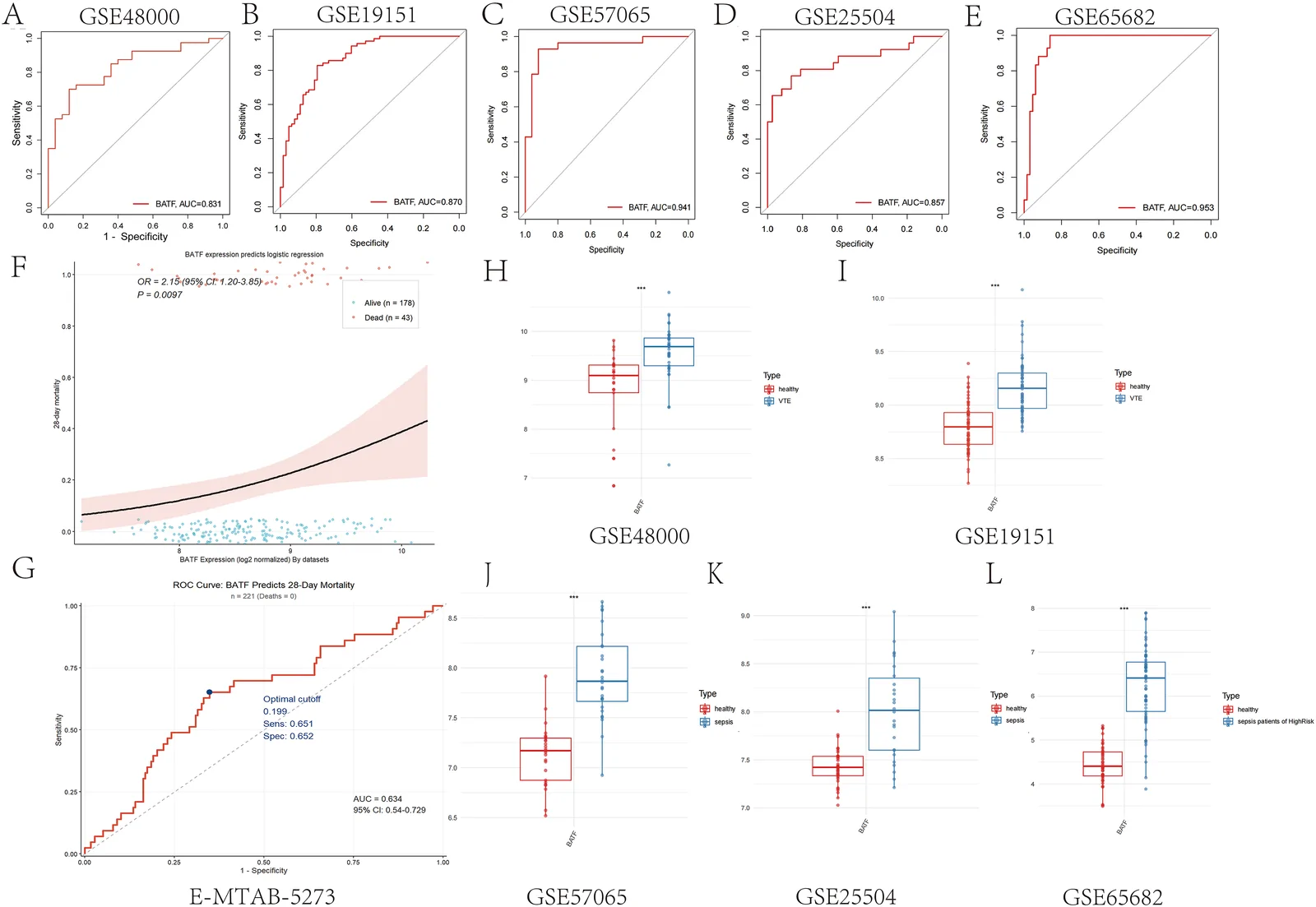
Diagnostic performance and validation of *BATF* expression across independent datasets. (A–E) Receiver operating characteristic (ROC) curves illustrating the diagnostic performance of BATF expression across different cohorts: (A) GSE48000 (AUC = 0.831), (B) GSE19151 (AUC = 0.870), (C) GSE57065 (AUC = 0.941), (D) GSE25504 (AUC = 0.857), and (E) GSE65682 (AUC = 0.953). (F, G) Logistic regression model analysis in the external validation dataset E-MTAB-5273: (F) Scatter plot showing the distribution of BATF mRNA levels and (G) ROC curve evaluating the model’s performance in the independent cohort. (H–L) Boxplots demonstrating the significant upregulation of BATF expression in sepsis and high VTE-risk patients compared to controls in: (H) GSE48000, (I) GSE19151, (J) GSE57065, (K) GSE25504, and (L) GSE65682. Statistical significance: ***p < 0.001.

In the GSE48000 dataset, *BATF* exhibited an area under the curve (AUC) of 0.831, while GSE19151 recorded an AUC of 0.870. For the sepsis datasets, *BATF* showed AUC values of 0.941 in GSE57065 and 0.857 in GSE25504. Remarkably, *BATF* also demonstrated good discriminatory performance in the GSE65682 dataset, with an AUC of 0.953.

To further assess the robustness and generalizability of *BATF*, we introduced E-MTAB-5273 as an external independent validation cohort that was not used in the original discovery or internal validation workflow. ROC analysis showed that *BATF* retained discriminatory ability in this external cohort, and logistic regression analysis further supported the association between *BATF* expression and the corresponding disease phenotype (Figs. 5F and 5G). These additional results strengthen the robustness of BATF as a candidate biomarker across independent datasets.

Overall, these results suggest that BATF may serve as a candidate biomarker associated with sepsis and VTE risk-related phenotypes across diverse patient cohorts. Boxplots further demonstrated that *BATF* was significantly upregulated in both sepsis and patients at high-risk of VTE across the datasets (Figs. 5H–5K). Notably, this upregulation trend was consistent in sepsis datasets at high-risk of VTE from the GSE65682 dataset as well (Fig. 5L). These findings reinforce the potential of BATF as a candidate marker, showing consistent changes in expression across different patient populations with sepsis and high VTE-risk phenotype. 

### *BATF* expression correlates with immune cell infiltration patterns

To characterize the immune landscape associated with *BATF* expression, we applied the CIBERSORT algorithm to estimate the relative proportions of 22 immune cell types in peripheral blood samples from the GSE57065 (sepsis) and GSE48000 (high VTE-risk) datasets. As shown in Figs. 6A and 6D, immune infiltration patterns differed markedly between disease and control groups. Specifically, M0 macrophages were significantly increased in both sepsis and VTE samples compared to healthy controls (Figs. 6B and 6E), suggesting shared features of immune activation.

**Figure 6:**
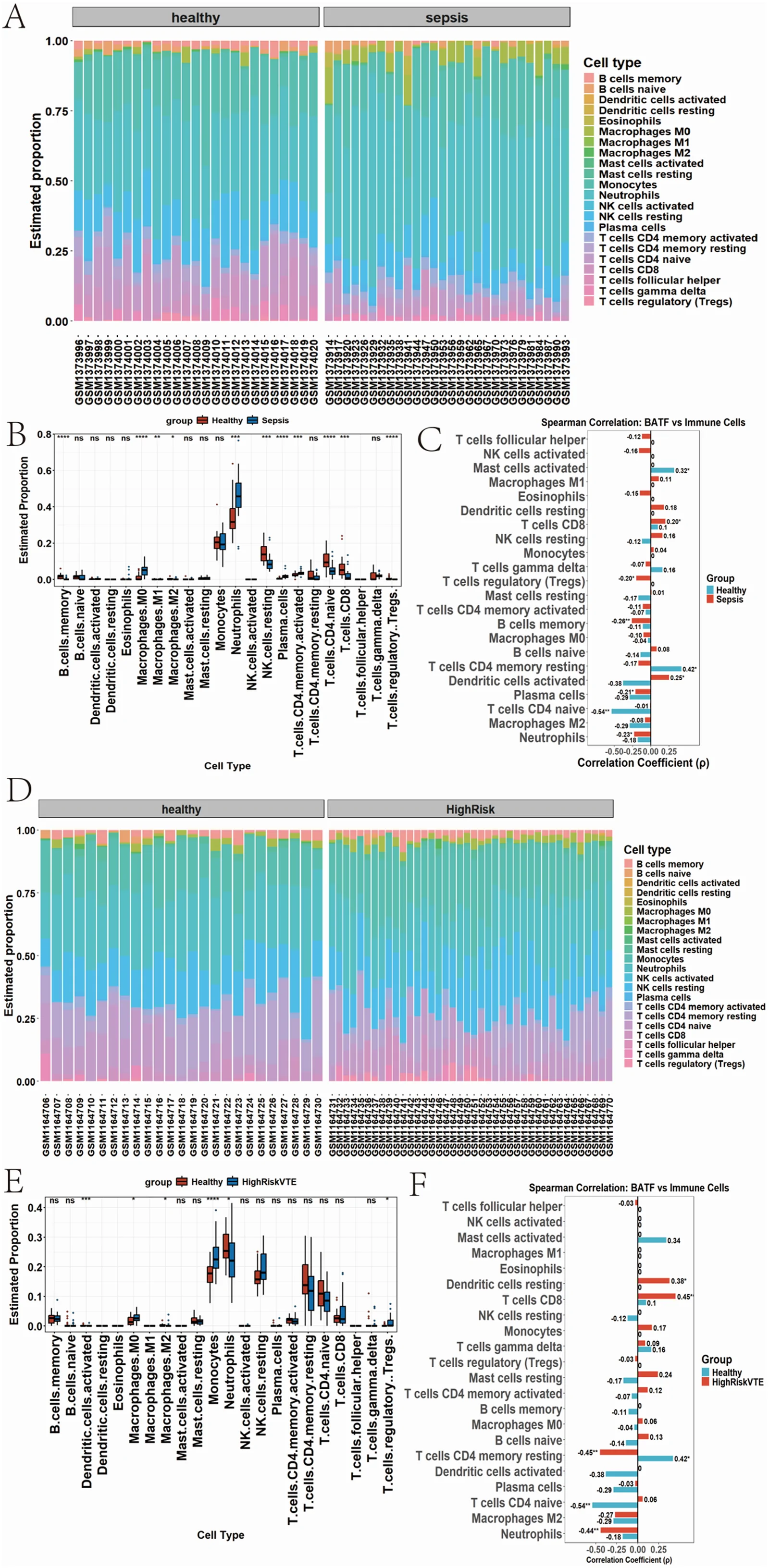
Immune cell infiltration and BATF-associated immunological profiling in sepsis and VTE datasets. (A, D) Relative proportions of 22 immune cell subtypes estimated by the CIBERSORT algorithm in the GSE57065 (sepsis) and GSE48000 (VTE risk) datasets, respectively. (B, E) Estimated proportions of individual immune cell subsets across disease and control groups; B and E show estimated cell proportions. (C, F) Correlation analysis between BATF expression and specific immune cell subsets (*e.g*., T cells, NK cells, neutrophils, macrophages) in the two datasets. Statistical significance: *p < 0.05, **p < 0.01, ***p < 0.001, ****p < 0.0001; ns, not significant.

Interestingly, neutrophils exhibited opposing trends: their infiltration was elevated in sepsis but markedly reduced in the VTE cohort, indicating distinct immunopathological processes. To further assess the association between *BATF* expression and immune cell subsets, we conducted Pearson correlation analyses. *BATF* expression was positively correlated with T cells and NK cells in both datasets (Figs. 6C and 6F). In addition, *BATF* showed a significant association with neutrophil levels in the VTE dataset, which was not observed in the sepsis group.

These results suggest that BATF may be involved in the regulation of immune cell composition, especially T cells, NK cells, and neutrophils, contributing to disease-specific immune responses in sepsis and high-risk VTE.

### Single-cell profiling confirms *BATF* enrichment in myeloid cell

This study also carried out a total of ten peripheral blood samples of single-cell transcriptome detection. After the quality control and the PCA dimensionality reduction, we got a total of 38,305 cells and 33,538 genes. And then, 19 clusters of cells were divided by clustering analysis (Fig. 7A). We annotated 10 cell types: T cells, myeloid cells, B cells, NK cells, plasma cells, erythroid cells, cycling cells, platelets, stromal cells, endothelial cells (Fig. 7B). Cycling cells represent actively proliferating cells identified based on the expression of cell cycle–related genes. We further analyzed the cellular distribution in sepsis datasets and healthy controls, as well as the expression patterns of *BATF* across various immune cell subsets (Fig. 7C). Our results revealed that *BATF* was significantly upregulated in T cells, NK cells, B cells, and myeloid cells, suggesting its potential involvement in immune regulation mechanisms. Additionally, the elevated expression of *BATF* in platelets implies its possible role in coagulation pathways (Fig. 7D).

**Figure 7:**
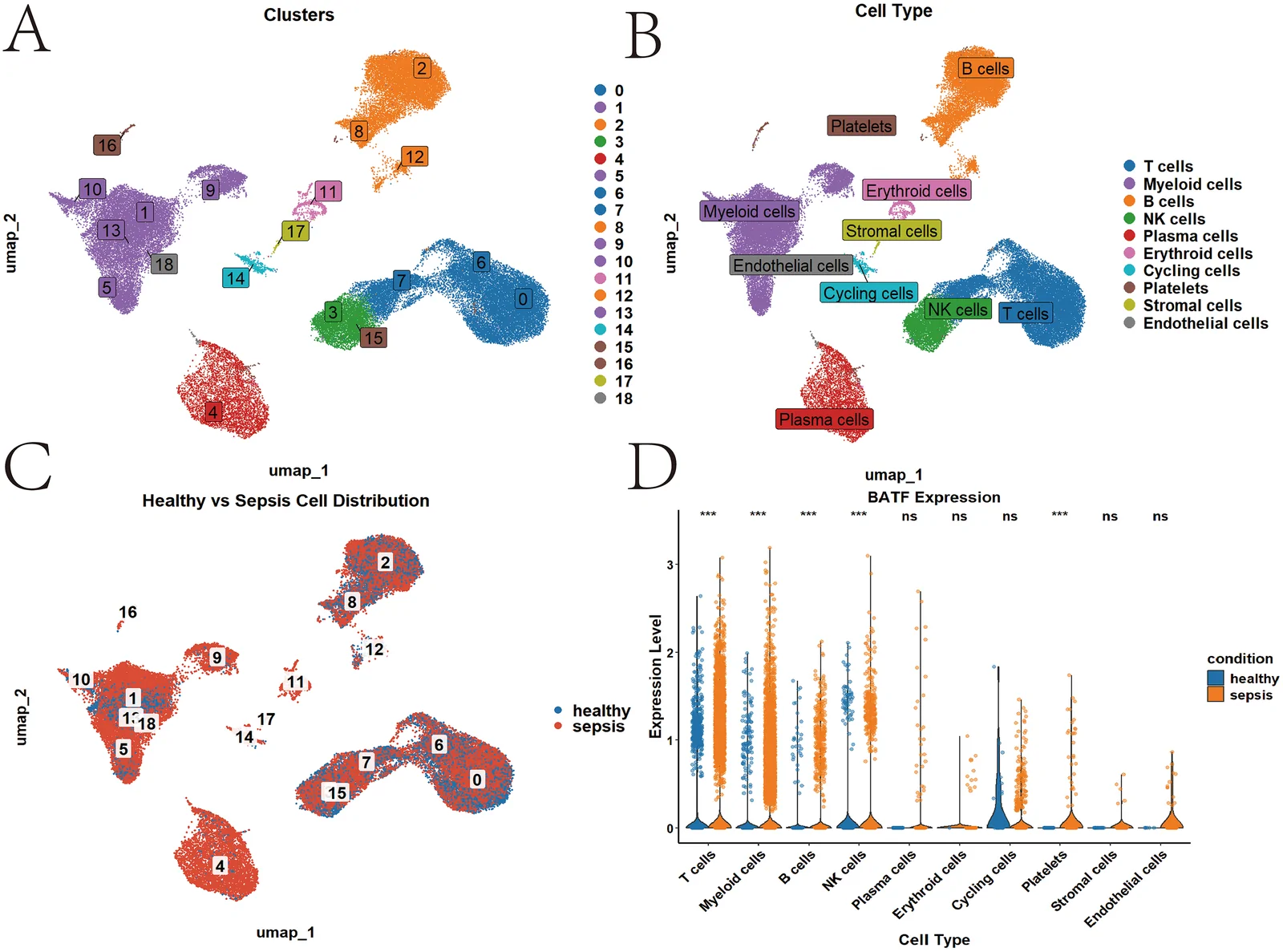
Single-cell RNA sequencing reveals BATF expression enriched in specific immune cell subsets in sepsis. (A) UMAP clustering of peripheral blood mononuclear cells (PBMCs) from sepsis and control samples, identifying 19 transcriptional clusters, Substantial overlap between healthy and sepsis-derived cells on the UMAP may reflect shared transcriptional states rather than complete cellular divergence. (B) Annotation of major immune cell types across clusters, including T cells, myeloid cells, B cells, NK cells, plasma cells, erythroid cells, cycling cells, platelets, stromal cells, and endothelial cells. (C) Distribution of BATF expression across different immune cell subsets from healthy controls and sepsis patients. (D) BATF expression across annotated cell types in control and sepsis samples. ***p < 0.001; ns, not significant.

### *BATF* expression is associated with 28-day mortality in sepsis patients with high VTE-risk phenotypes

Survival curves reveal a significant correlation between *BATF* expression and the survival prognosis of sepsis patients at high risk for venous thromboembolism (VTE) (Fig. 8A). Survival analysis in GSE65682 evaluated 28-day mortality among sepsis patients stratified by BATF expression. Because this dataset does not contain adjudicated VTE outcomes, the analysis should be interpreted as assessing sepsis-related short-term prognosis rather than VTE-related mortality or thrombosis prediction (Table S3). The clinical factors included in the analysis were age, gender, presence of pulmonary infections (either community-acquired or hospital-acquired), history of diabetes mellitus (type not specified in the original dataset metadata), ICU-acquired infections, and abdominal infections.

**Figure 8:**
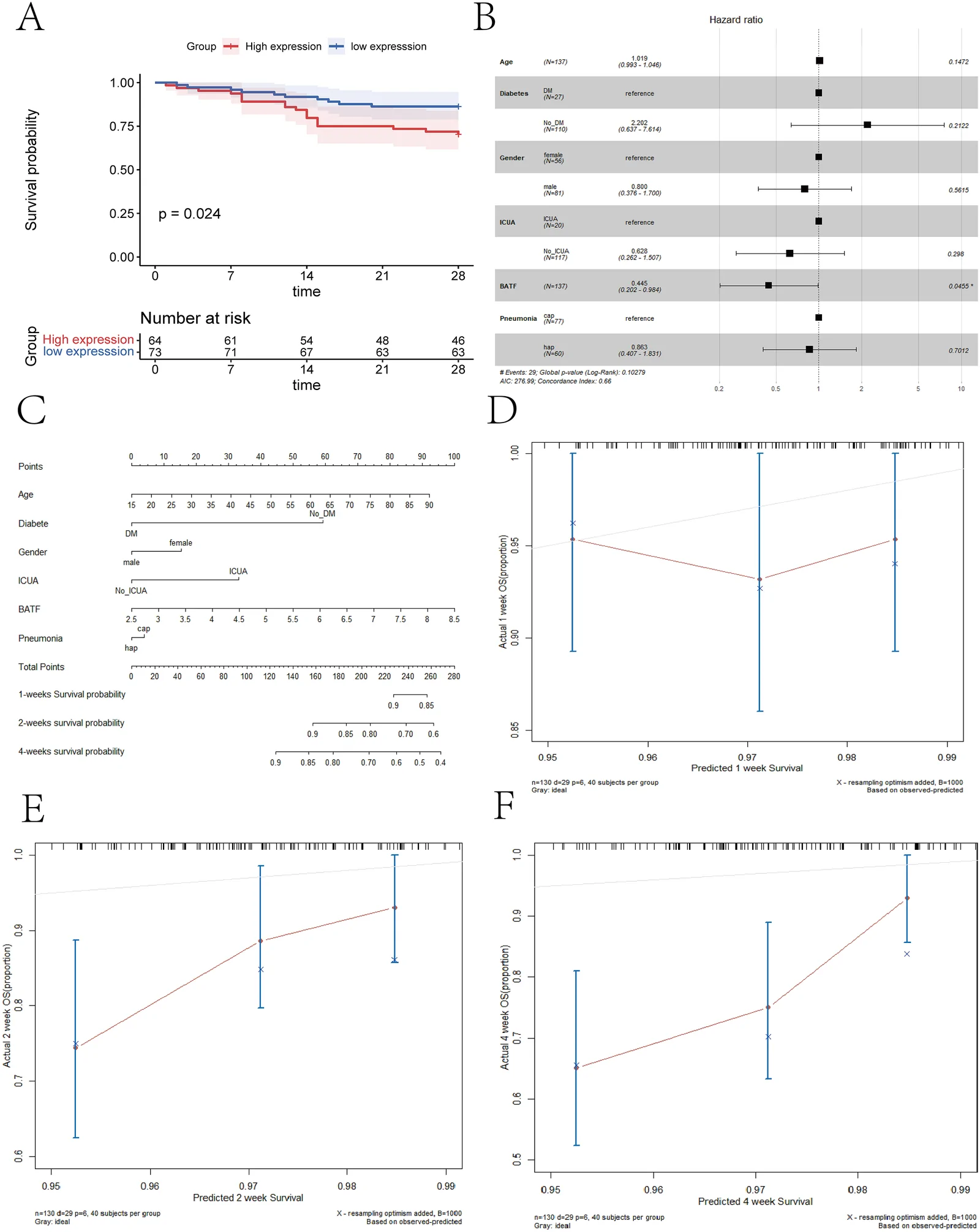
Clinical correlational analysis. (A) Survival analysis of BATF in 28 days by cutoff method. (B, C) Forest plot and nomogram plot for multivariate Cox regression analysis. (D–F) 1-, 2-, 4-weeks survival rate calibration curve.

The Cox analysis results confirmed a significant correlation between BATF and survival (Fig. 8B). To enhance the interpretability of these findings, we constructed a nomogram (Fig. 8C) to estimate survival probability at 1-, 2-, and 4-week intervals (Figs. 8D–8F). The nomogram showed only modest prognostic discrimination (C-index = 0.66). This indicates that the model has limited clinical utility as a standalone prognostic tool. Therefore, BATF should be considered an exploratory adjunctive marker for 28-day mortality risk stratification in sepsis rather than an independent clinical decision-making tool.

### Experimental validation of BATF expression and function in sepsis and thromboinflammatory models

To validate the expression levels of BATF in sepsis, we established a sepsis model in THP-1-derived macrophages by differentiating the cells with phorbol 12-myristate 13-acetate (PMA) followed by lipopolysaccharide (LPS) stimulation. For the endothelial inflammation model relevant to thromboinflammatory responses, we utilized the HUVEC cell line, treating it with LPS for 24 h. RT-qPCR analysis confirmed that *BATF* expression levels were consistent with our bioinformatics dataset, showing a significant upregulation in both the THP-1-derived sepsis model and the HUVEC endothelial inflammation model (Figs. 9A and 9C). Furthermore, validation using patient blood samples confirmed that *BATF* expression was significantly elevated in sepsis patients and sepsis patients with high risk of VTE compared to healthy controls (Figs. 9B and 9D), consistent with the observed transcriptomic association. Longitudinal analysis further revealed that *BATF* mRNA levels peak at Day 1 and significantly decrease by Day 3 in survivors, suggesting its primary involvement in the early inflammatory activation phase of sepsis (Fig. S1).

**Figure 9:**
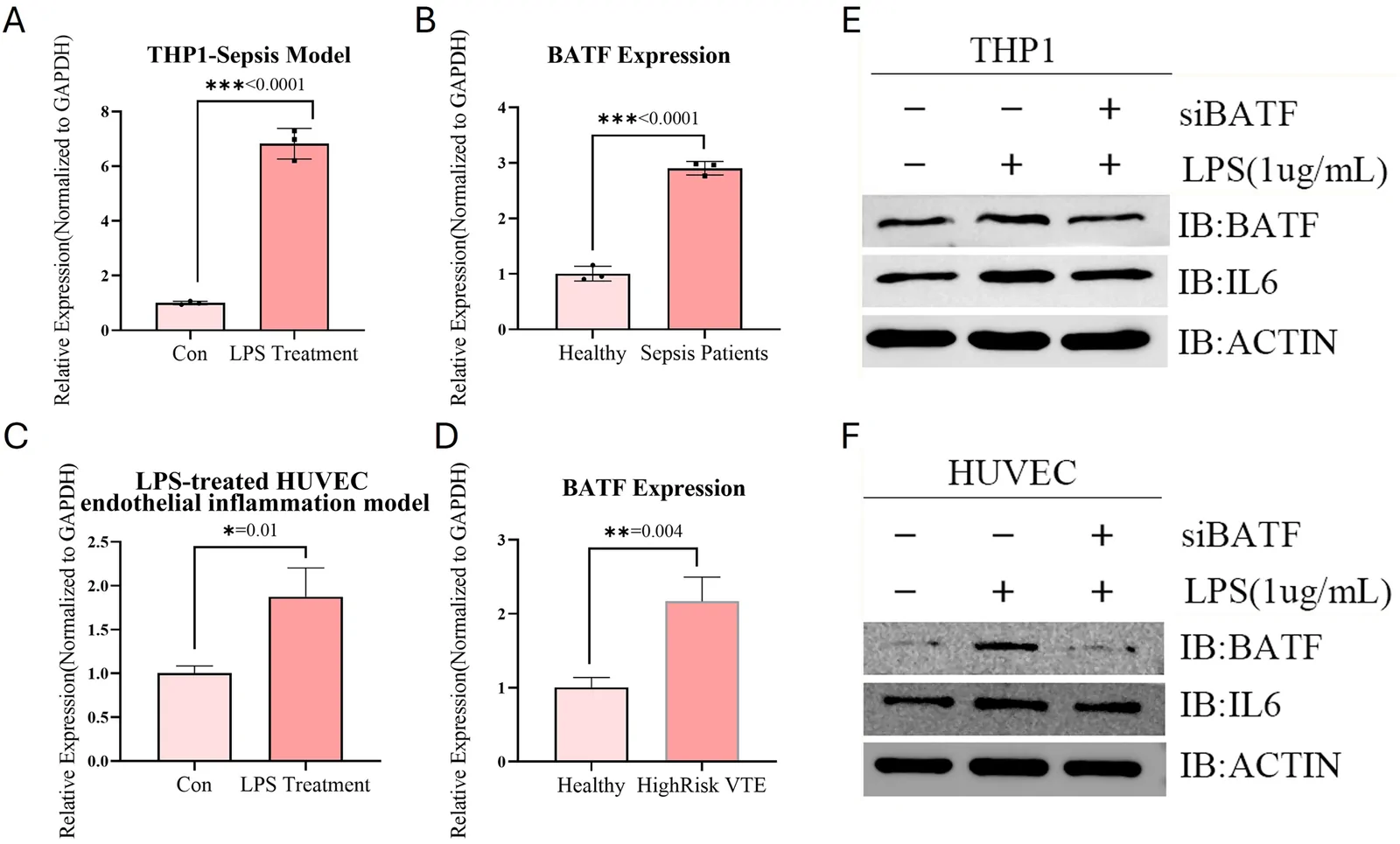
Quantitative PCR (qPCR) and WB analysis. (A) Expression of BATF in the THP-1 Cell Model. (B) Expression of BATF in peripheral blood of sepsis patients. (C) Expression of BATF in the LPS-treated HUVEC endothelial inflammation model. (D) Expression of BATF in peripheral blood of high risk VTE patients. (E, F) Western blot analysis showing BATF knockdown and its effect on IL6 protein expression in THP-1-derived macrophages and HUVEC cells after LPS stimulation. For panels A–D, *p = 0.01, **p = 0.004, and ***p < 0.0001, as labeled in the panels.

To further explore the potential functional relevance of BATF in inflammatory signaling, we performed siRNA mediated *BATF* knockdown followed by Western blot analysis. BATF protein expression increased after LPS stimulation in both THP-1-derived macrophages and HUVEC cells. *BATF* knockdown effectively reduced BATF protein levels and was accompanied by altered IL6 protein expression (Figs. 9E and 9F). These findings provide preliminary experimental support for the involvement of BATF in inflammatory responses associated with sepsis and thromboinflammatory conditions.

## Discussion

Sepsis and VTE are interrelated conditions with significant morbidity and mortality, especially in critically ill patients. Despite advances in understanding their pathophysiology, shared molecular mechanisms remain elusive. In this study, we employed bioinformatics and experimental validation approaches to identify BATF as a candidate marker associated with disease status, high VTE risk phenotypes, and 28-day mortality in sepsis. However, because confirmed VTE events were not available, its value for VTE diagnosis or thrombosis prediction remains unproven.

### BATF as a candidate marker associated with sepsis and high VTE-risk phenotypes

Our study identified BATF as a candidate marker showing discriminatory performance across multiple sepsis- and VTE risk- related datasets. The AUC values ranging from 0.831 to 0.953, suggest that BATF expression may help distinguish disease- or risk-score-defined phenotypes from controls. However, because these analyses were based on sepsis status and VTE risk-score-defined phenotypes rather than confirmed VTE events, BATF should not be interpreted as a diagnostic biomarker for VTE.

Additionally, survival analysis showed that *BATF* expression was associated with 28-day mortality in sepsis patients with high VTE-risk phenotypes. The nomogram developed using Cox regression analyses showed only modest predictive performance (C-index = 0.66). Therefore, its clinical utility as a standalone prognostic model is limited. BATF may provide complementary prognostic information when combined with established clinical predictors, but it should not be interpreted as an independent prognostic or clinical decision-making tool.

Thus, BATF should be considered an exploratory candidate marker associated with disease status, high VTE-risk phenotypes, and 28-day mortality in sepsis, rather than a validated biomarker for VTE diagnosis or thrombosis prediction.

### Involvement of BATF in immune signaling and inflammation

Functional enrichment analyses revealed that BATF is involved in critical biological processes such as immune cell activation (Lewis et al., 2011; Sun et al., 2023; Übel et al., 2014; Zhang & Jiang, 2024), cytokine production, and immune signaling pathways, including PD-L1 expression and Th17 cell differentiation (Peng et al., 2025; Pham et al., 2023; Qiu et al., 2017; Wang et al., 2025). These findings align with BATF’s established role in immune regulation and suggest that BATF expression is associated with immune dysregulation observed in sepsis and VTE-related conditions (Catar et al., 2021; Guo et al., 2021; Liu et al., 2020; Seo et al., 2021).

Single-cell RNA sequencing (scRNA-seq) analysis further illustrated that *BATF* expression is enriched in myeloid cell, two critical cell types in infection and innate immunity. This dual expression pattern suggests that BATF may serve as a marker for immune activation in sepsis rather than a confirmed mechanistic driver.

### BATF-immune cell interactions reveal differential infiltration patterns

Immune infiltration analysis demonstrated *BATF*'s context-specific relationship with immune cell populations. In sepsis, *BATF* showed a positive correlation with neutrophil infiltration, while in VTE, the correlation was negative. This differential suggests that *BATF* expression may reflect distinct immune responses in these conditions. Additionally, *BATF* was positively correlated with M2 macrophage infiltration in both datasets, further supporting its involvement in immune regulation.

These findings suggest that *BATF* expression may reflect immune-thrombotic alteration relevant to sepsis and VTE risk (Guo et al., 2021; Liu et al., 2020; Peng et al., 2025; Pham et al., 2023; Wang et al., 2025). However, given the observational nature of this study, further experimental investigations are required to determine whether BATF directly regulates immune-thrombotic interactions.

### Study limitations and future prospects

While our findings demonstrate BATF’s potential as a biomarker, there are limitations to this study. First, the datasets analyzed were derived from public repositories, and further validation in independent and prospective cohorts is essential. The reliance on publicly available datasets introduces potential biases related to sample selection, processing techniques, and variations in clinical metadata, which may impact the reproducibility of our findings. Future studies should integrate multi-center, real-world patient data to enhance generalizability.

Second, the heterogeneity in patient cohorts, including variations in infection types, comorbidities, and treatments, may influence BATF’s generalizability. Different underlying conditions such as bacterial *vs*. viral infections, or varying anticoagulation regimens, could introduce confounding effects in BATF expression patterns. Stratification of patient subgroups based on these factors in future analyses could provide deeper insights into BATF’s role in specific clinical contexts.

Third, although scRNA-seq and immune infiltration analyses provided insights into BATF’s cellular and molecular roles, experimental studies are needed to elucidate its precise mechanisms. The exact signaling pathways by which BATF influences immune-thrombosis remain unclear, necessitating functional assays such as CRISPR-based knockdown models or *in vivo* sepsis-induced thrombosis models to dissect BATF’s biological role in linking immune response and coagulation abnormalities.

Additionally, our study was limited by the lack of transcriptomic datasets containing sepsis patients with adjudicated VTE events. Instead, this study integrates sepsis datasets with cohorts representing individuals at high risk for VTE. Therefore, BATF should be interpreted as a biomarker associated with increased VTE risk rather than a confirmed predictor of thrombosis in sepsis patients. This limitation reduces the strength of causal inference and restricts the direct linkage of our findings to sepsis-associated VTE events. Accordingly, the present findings identify BATF as a candidate associated with VTE risk factors and risk-score-defined phenotypes, rather than a biomarker validated for VTE diagnosis, confirmed thrombotic events, or thrombosis prediction. Future studies incorporating well-characterized cohorts with adjudicated VTE outcomes are required to validate these findings and strengthen their clinical relevance (Fleischmann-Struzek & Rudd, 2023; Singer et al., 2016).

Future research should focus on validating BATF’s biomarker potential through prospective clinical trials. Investigating BATF-associated immune and coagulation pathways in preclinical models could provide insights into its preliminary functional relevance. Further studies should explore the interaction of BATF with immune and coagulation pathways to better understand its association with sepsis-related thromboinflammatory responses. Moreover, integrating transcriptomics with proteomics and metabolomics data may uncover broader molecular signatures associated with sepsis-induced thrombosis, paving the way for precision medicine approaches in critically ill patients.

## Conclusion

In summary, our study supports BATF as a candidate biomarker associated with sepsis, high VTE-risk phenotypes, and 28-day mortality. Because this study relied on VTE risk scores rather than adjudicated VTE events, BATF should not be interpreted as a validated biomarker for confirmed VTE diagnosis or thrombosis prediction. BATF expression was associated with immune and coagulation-related alterations, but its biological role remains to be fully established. These findings support further prospective validation and exploratory investigation: (1) validating BATF’s biomarker potential through prospective clinical trials, (2) exploring BATF-associated immune and coagulation pathways in future mechanistic studies, and (3) developing dynamic monitoring tools to assess BATF expression as a potential biomarker of disease progression. Collectively, these efforts may contribute to more precise management strategies for critically ill patients.

## Supplemental Information

10.7717/peerj.21798/supp-1 Supplemental Information 1 Illustrates the study workflow for identifying BATF as a shared biomarker associated with sepsis and venous thromboembolism, integrating transcriptomic analysis, machine-learning-based screening, immune profiling, and experimental validation.

10.7717/peerj.21798/supp-2 Supplemental Information 2 MIQE checklist.

10.7717/peerj.21798/supp-3 Supplemental Information 3 Raw data for qPCR.

10.7717/peerj.21798/supp-4 Supplemental Information 4 R code for analysis.

10.7717/peerj.21798/supp-5 Supplemental Information 5 Longitudinal analysis of BATF gene expression in sepsis survivors. Boxplot illustrating the temporal expression patterns of *BATF* mRNA in peripheral blood samples from the GSE95233 dataset. The analysis includes healthy controls, sepsis survivors at Day 1, and the same survivors at Day 3 post-enrollment. *BATF* expression is significantly upregulated at both Day 1 and Day 3 compared to healthy controls (*p* < 0.001). In addition, BATF expression shows a significant decrease from Day 1 to Day 3 within the survivor group. Data are presented as log2 - transformed expression values. Statistical significance was determined by one-way ANOVA followed by Tukey’s *post hoc* test. **p < 0.01; ***p < 0.001.

10.7717/peerj.21798/supp-6 Supplemental Information 6 The information of sepsis patients.

10.7717/peerj.21798/supp-7 Supplemental Information 7 The information of High Risk VTE patients. Clinical characteristics of sepsis patients at high risk for venous thromboembolism (VTE).

10.7717/peerj.21798/supp-8 Supplemental Information 8 Univariate and multivariate Cox analysis.

## Additional Information and Declarations

### Data Availability

The following information was supplied regarding data availability:

The data and code are available in the Supplemental Files.

The data is available at Array Express: Katie Burnham (2017). Microarray transcriptomic profiling of patients with sepsis due to faecal peritonitis and pneumonia to identify shared and distinct aspects of the transcriptomic response (discovery cohort). BioStudies, E-MTAB-5273. Retrieved from https://www.ebi.ac.uk/biostudies/ArrayExpress/studies/E-MTAB-5273.

The sequence data is available at NCBI: GSE19151, GSE65682, GSE48000, GSE57065, GSE95233, GSE25504.
