# Supplementary material for: Basic leucine zipper ATF-like transcription factor (BATF) as a candidate marker associated with venous thromboembolism risk factors in sepsis: an integrated bioinformatics and machine learning analysis
Source: PeerJ. 2026 Oct 20;14:e21798. doi: 10.7717/peerj.21798 (PMC13693607; doi:10.7717/peerj.21798)
Supplement: Supplementary file 1 [file peerj-14-21798-s001.pdf]

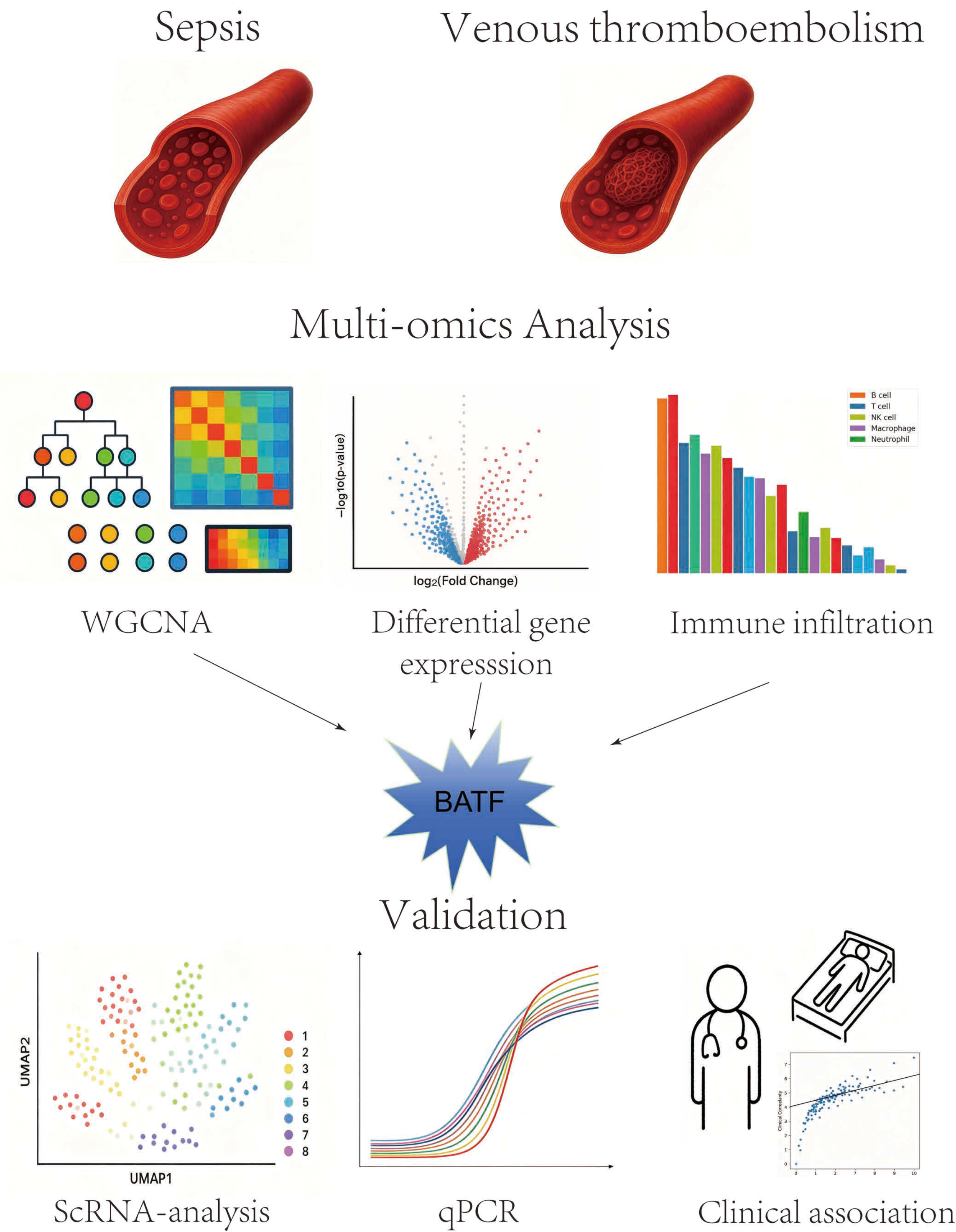

Sepsis
Venous thromboembolism
Multi-omics Analysis
B cell
T cell
NK cell
Macrophage
Neutrophil
−log10(p-value)
log2(Fold Change)
WGCNA
Differential gene expresssion
Immune infiltration
BATF
Validation
UMAP2
UMAP1
1
2
3
4
5
6
7
8
ScRNA-analysis
qPCR
Clinical association
