# Supplementary material for: Basic leucine zipper ATF-like transcription factor (BATF) as a candidate marker associated with venous thromboembolism risk factors in sepsis: an integrated bioinformatics and machine learning analysis
Source: PeerJ. 2026 Oct 20;14:e21798. doi: 10.7717/peerj.21798 (PMC13693607; doi:10.7717/peerj.21798)
Supplement: Supplementary file 2 [file peerj-14-21798-s006.docx]

**Table 1.The information of sepsis patients**

| Information of Patients | | | SOFA | | | | | Presence or absence of infection |
| --- | --- | --- | --- | --- | --- | --- | --- | --- |
| Number | Age | Gender | 0 | 1 | 2 | 3 | 4 |  |
|  |  |  |  |  |  |  |  |  |
| 7250216 | 64 | Female | MAP≥70mmHg | oxygen index＜400mmHg | / | Platelet count(1×109)＜50 | / | √ |
| 7250820 | 51 | Male | MAP≥70mmHg | / | oxygen index＜200&mechanical ventilation | / | / | √ |
| 7255848 | 76 | Male | / | / | Dobutamine infusion at 5 μg/(kg*min) for more than 1 hour | Creatinine≥440&Urinary volume＜500ml/d | / | √ |
