# Supplementary material for: Basic leucine zipper ATF-like transcription factor (BATF) as a candidate marker associated with venous thromboembolism risk factors in sepsis: an integrated bioinformatics and machine learning analysis
Source: PeerJ. 2026 Oct 20;14:e21798. doi: 10.7717/peerj.21798 (PMC13693607; doi:10.7717/peerj.21798)
Supplement: Supplementary file 3 [file peerj-14-21798-s007.docx]

**Table2.The information of High Risk VTE patients**

| Information of Patients | | | VTE assessment | | | | |
| --- | --- | --- | --- | --- | --- | --- | --- |
| Number | Age | Gender | 1 Score | 2 Scores | 3 Scores | 5 Scores | The risk level of VTE |
|  |  |  |  |  |  |  |  |
| 7289483001 | 78 | Male | / | Bedridden＞3days | ＞75 yeas old | / | HighRisk(5 Scores) |
|  |  |  |  |  |  |  |  |
| 7289036002 | 53 | Male | 41-60 years old | Bedridden＞3days | Major open surgery | / | HighRisk(6 Scores) |
|  |  |  |  |  |  |  |  |
| 7289020001 | 44 | Male | 41-60 years old | Central Venous Catheter | / | Fracture of the hip, pelvis, or lower extremities | HighRisk(8 Scores) |
|  |  |  |  |  |  |  |  |
