# Supplementary material for: Basic leucine zipper ATF-like transcription factor (BATF) as a candidate marker associated with venous thromboembolism risk factors in sepsis: an integrated bioinformatics and machine learning analysis
Source: PeerJ. 2026 Oct 20;14:e21798. doi: 10.7717/peerj.21798 (PMC13693607; doi:10.7717/peerj.21798)
Supplement: Supplementary file 4 [file peerj-14-21798-s008.docx]

**Table 3. Univariate and multivariate Cox analysis**

| **Variables** | **Univariate** **analysis** | | | **Multivariate Analysis** | | |
| --- | --- | --- | --- | --- | --- | --- |
|  | **95%Cl** **of** **HR** | **HR** | **P-value** | **95%Cl** **of** **HR** | **HR** | **P-value** |
| Age | 0.9892~1.0400 | 1.014 | 0.271 | 0.9932~1.0465 | 1.0195 | 0.1472 |
| Gender | 0.4058~1.754 | 0.8437 | 0.649 | 0.3763~1.7001 | 0.7998 | 0.5615 |
| Diabetes | 0.6928~7.563 | 0.8281 | 0.174 | 0.6371~7.6145 | 2.2025 | 0.2122 |
| ICUA | 0.2463~1.351 | 0.5768 | 0.205 | 0.2620~1.5073 | 0.6284 | 0.2980 |
| Pneumonia | 0.4693~2.057 | 0.9826 | 0.963 | 0.4067~1.8314 | 0.8630 | 0.7012 |
| BATF | 0.1908~0.8833 | 0.4106 | 0.0228* | 0.2015~0.9838 | 0.4453 | 0.0455* |
